# Mining Proteome Research Reports: A Bird’s Eye View

**DOI:** 10.3390/proteomes9020029

**Published:** 2021-06-10

**Authors:** Jagajjit Sahu

**Affiliations:** National Centre for Cell Science (NCCS), NCCS Complex, Pune University Campus, Ganeshkhind Road, Pune 411007, Maharashtra, India; sahujagajjit@gmail.com; Tel.: +91-9508087443

**Keywords:** proteome, NLP, scientometrics, text mining, bio-concepts, gene–gene network

## Abstract

The complexity of data has burgeoned to such an extent that scientists of every realm are encountering the incessant challenge of data management. Modern-day analytical approaches with the help of free source tools and programming languages have facilitated access to the context of the various domains as well as specific works reported. Here, with this article, an attempt has been made to provide a systematic analysis of all the available reports at PubMed on Proteome using text mining. The work is comprised of scientometrics as well as information extraction to provide the publication trends as well as frequent keywords, bioconcepts and most importantly gene–gene co-occurrence network. Out of 33,028 PMIDs collected initially, the segregation of 24,350 articles under 28 Medical Subject Headings (MeSH) was analyzed and plotted. Keyword link network and density visualizations were provided for the top 1000 frequent Mesh keywords. PubTator was used, and 322,026 bioconcepts were able to extracted under 10 classes (such as Gene, Disease, CellLine, etc.). Co-occurrence networks were constructed for PMID-bioconcept as well as bioconcept–bioconcept associations. Further, for creation of subnetwork with respect to gene–gene co-occurrence, a total of 11,100 unique genes participated with mTOR and AKT showing the highest (64) number of connections. The gene p53 was the most popular one in the network in accordance with both the degree and weighted degree centrality, which were 425 and 1414, respectively. The present piece of study is an amalgam of bibliometrics and scientific data mining methods looking deeper into the whole scale analysis of available literature on proteome.

## 1. Introduction

The booming of omics technologies has notably influenced almost all the realms of life science. These technologies have paved boundless ways for the extraction of much hidden and intricate information, which are pivotal to accelerate many scientific endeavors. On the contrary, the massive amount of data has created the Big Data issue, and to manage such data, efficient data analytics approaches are required. These kinds of technologies have resulted in exponential growth in the scientific literature too, along with other high-throughput data. Text mining is an emerging and widely used approach to extract meaningful information and bridge them across different texts irrespective of their sources such as scientific articles or blogs or other similar sources. Implementation of the powerful algorithm as well as technologies such as artificial intelligence (AI), machine learning (ML), deep learning, etc., aided with programming languages have been a common practice in recent years. These have also been reflected in the form of several reports published during the last few decades, which have considered text mining for providing systematic reviews, scientometric analysis, extraction of various bioconcepts and constructing knowledge graphs through scientific relations, etc. [1,2,3,4].

Natural Language Processing (NLP) dispense umpteen techniques to mine scientific text [5,6]. It is a hectic task to mine the text data as it is unstructured and ambiguous in nature. However, in the last few years, there have been remarkable improvements in text mining techniques. The emergence of a huge number of biological corpora and dictionaries have been boosting these techniques to cater the bioconcept identification. Named Entity Recognition (NER) is one of the foremost subtasks of text mining, which contributes primely to information extraction [7]. NER aims to capture all entities (commonly known as bioconcepts in biology) and their types such as individual genes or gene families, cell lines, species, etc., along with their occurrences in a given text [8]. Usually, the next effort after NER is to establish relations between the bioconcepts. This is a bit dodgy, as often it attempts to capture the sentiment or context of the text. However, a simpler way to start with connecting bioconcepts is to consider the co-occurrences. These networks can again be enriched with additional information and meta-data to build a knowledge graph (KG).

The current study targets to forge KGs for various bioconcept classes, thereby drawing a picture of the research works that have been published in the field of proteome analysis. The proteome is defined as the whole/all protein in a cell, tissue, or organ [9]. It is well-known that the study of proteome contributes to the maximum to understand the functional make-up of a particular organism at a particular instant [10]. Despite the advancements in high-throughput technologies, it is a herculean task due to various post-translational modifications, vast varieties with different levels of expressions, etc. [11,12]. Adding to the misery, the difficulty level goes up due to the non-targeted nature of investigations in the case of proteomics analysis. Recent improvements in text analytics have been successful to collate available information up to a great extent. With regard to proteome literature mining, the number of reports published is still scanty. The current study targets to bring forth an overall understanding of the past and current scenario of publications related to proteome with an absolute extraction of the important bioconcepts from the available reports.

## 2. Materials and Methods

### 2.1. Collection of Data

To access the reports related to the term “Proteome”, it was used as a Mesh term and searched on PubMed. The English language filter was selected along with a custom date up to 31 December 2020. The date range was changed to perform batch downloads, as PubMed did not allow downloads of more than 10,000 records at once. The PubMed Identifiers (PMIDs) for all the retrieved records were then saved into separate files. The citation data in both text and bibtex formats were also downloaded and saved into separate files. In addition, PMIDs were downloaded for all the Mesh subheadings separately. Each subheading was saved into individual files and, wherever necessary (more than 10,000 records), the records were downloaded in batch and merged. Most of the further processing is assisted by in-house R scripts (https://github.com/sahujagajjit/PubTator2Entity accessed on 9 June 2021).

### 2.2. Preprocessing and Scientometric Analysis

The PMIDs, as well as citation files, were merged into single files, respectively, with the help of R scripts. The R package bibliometrix was used to convert the PubMed citation data into simple data frames on the R console [13]. The publication years and their respective frequencies were calculated from the data frame and the year wise trend of publication quantities were accessed and plotted as a bar diagram using the ggplot2 package [14]. Next, the numbers of PMIDs collected for each subheading were checked and tried to build a visualization for the significant intersections between them. A powerful R package UpSetR was used for the visualization of intersections [15]. An in-house R function was developed to prepare the input file for UpSetR. The input file had all the unique PMIDs from all the subheadings as the first column and all other columns represented the subheadings. The rows were basically the PMIDs for the first column and the presence and absence of the PMID in corresponding subheadings as 1 and 0, respectively.

### 2.3. Keyword Mining

The widely used tool VOSviewer was used for the mining of the Mesh keywords for all the articles using the merged PubMed citation files in bibtext format [16]. Only the Mesh keywords option was chosen for the plot and the minimum frequency of the keyword was kept as 5. Network and density visualizations were constructed for the most frequent keywords.

### 2.4. Mining of Bioconcepts

The idea of mining bioconcepts was to extract possible entity information which can help in creating knowledge graphs (KG). Since the main source of data collection was PubMed, entity mining was carried out using PubTator Central [17]. PubTator Central (PTC) is a well-maintained and curated service for automated annotation of bioconcepts from the text. All the selected PMIDs were uploaded onto PubTator’s Collections Manager and allowed to annotate. The annotations were downloaded in the Pubtator format to the local system and the entity information was extracted onto a comma-separated value file. Next, 2 graph objects were created for PMID-EntityID and EntityID-EntityID associations, respectively. The graph objects were exported to the local system and further imported onto the Gephi tool for calculating network properties and visualization [18]. A bioconcept co-occurrence network was created from the available bioconcept information and saved in a structure equivalent to the edge file so that it can directly be imported to Gephi. The essential network properties such as average degree, average weighted degree, clustering coefficient and modularity were calculated. To provide a better scientific perspective, the gene–gene network was created by extracting all the gene entities from the bioconcept co-occurrence network. The network was studied with the help of network properties, and participation information for the top 10 genes were also extracted.

## 3. Results

**Figure 1:**
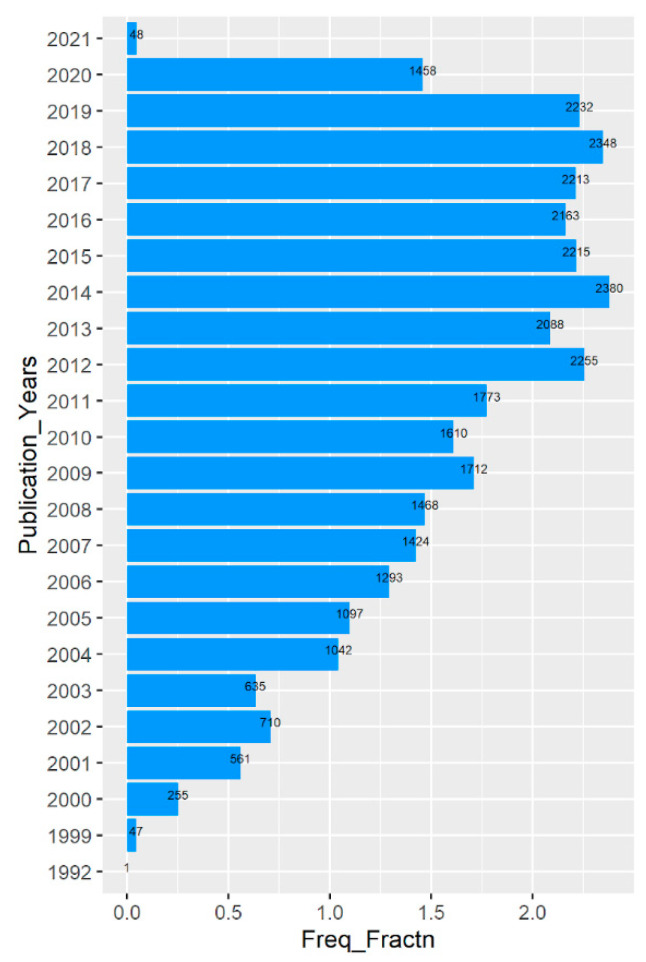
Year-wise publication trend. Freq_Fractn represents the actual no of publications/1000 and the actual no of publications are presented on the top of the bars.

Initially, a total of 33,028 records were retrieved and downloaded by using Proteome as the Mesh term with filters for English language and date up to 31 December 2020. A basic scientometrics analysis provided that the highest number of publications was in the year 2014 with 2380 articles (Table 1, Figure 1). The year ranged to 2021 despite the date filter being 31 December 2020 because of the consideration of the actual publication year for plotting. The highest publishing journal in the list was found to be Proteomics, with 2500 articles among a total of 1745 journals. Though the extraction of country information from the affiliation was not possible for all the records, an overall prediction suggests that the United States was in the lead.

**Figure 2:**
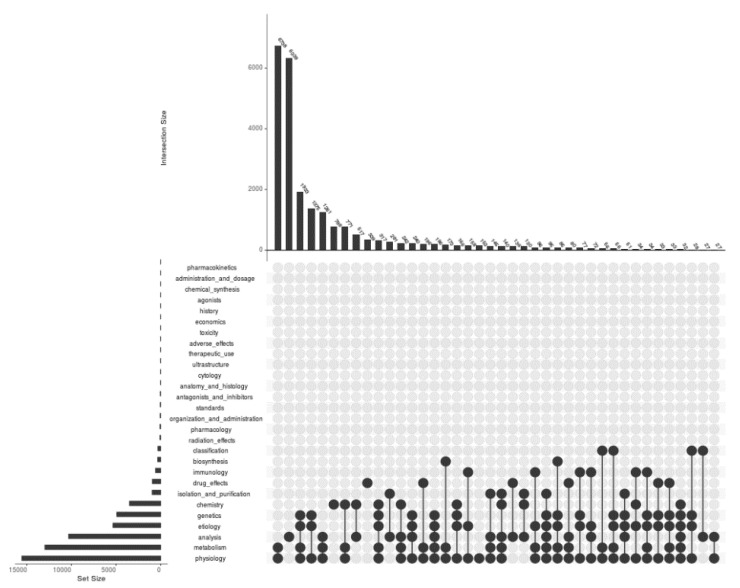
A plot for visualizing the intersections of PMIDs between 28 types of Mesh subheadings for the Mesh term Proteome. The input file contained a total of 24,350 unique PMIDs that are present under at least one of the subheadings. The matrix was of 24,350 rows and 28 columns for all subheadings (with the first column containing 24,350 PMIDs). All 28 subheadings have been labelled in the graph along with the number of PMIDs as a bar plot to the left. Right to the labels, there are black and grey circles representing the sets participating in the intersection and not participating, respectively. The black lines connecting the black circles represent the exclusive intersections.

Categorizing articles under various research areas is one of the beautiful outcomes of modern text mining procedures. There were 28 Mesh subheadings under the Proteome Mesh term for which the PMIDs were downloaded and saved into files. The intersection profiles with respect to PMIDs under all 28 subheadings have been presented in Figure 2. The highest number of PMIDs was found in the case of physiology with a value of 15,566 and with 3, pharmacokinetics is the subheading containing the lowest. The results identified the largest intersection of 6738 between the subheadings physiology and metabolism. Metabolism contains the second-highest number of PMIDs, followed by physiology, which is 12,979. The highest number of subheadings participating in a single intersection is 6 and occurred for two intersections; with 96 and 32 number of PMIDs at the 25th and 37th bar, respectively. The subheadings that did not participate in the intersections are 17 in number and are pharmacokinetics, administration and dosage, chemical synthesis, agonists, history, economics, toxicity, adverse effects, therapeutic use, ultrastructure, cytology, anatomy and histology, antagonists and inhibitors, standards, organization and administration, pharmacology, and radiation effects.

**Figure 3:**
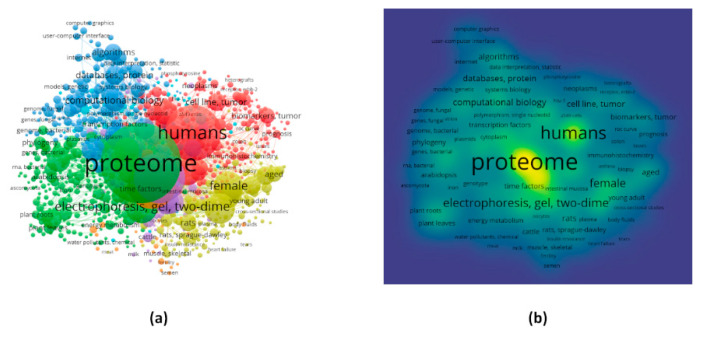
Visualization of the Mesh keywords for all the articles with the help of VOSviewer. (**a**) and (**b**) are network and density visualizations for the frequent keywords, respectively. Keeping the co-occurrence links in mind, the number of keywords for the visualization was limited to 1000.

The analysis in VOSviewer could able to extract 12,933 keywords, among which 5389 met the cut-off that is to have a minimum frequency of 5. The keywords co-occurrence network consisted of 7 clusters with 266, 252, 167, 144, 80, 58, and 33 keywords, respectively (Figure 3a). A table has been provided for the top 20 frequent keywords between 1000 keywords (Table 2). The highest frequency was found to be 32,273 for the keyword “proteome” belonging to the second cluster. The Mesh keyword in the second position was “humans” with a frequency of 15,686 followed by “animals”, “electrophoresis, gel, two-dimensional”, “mass spectrometry”, etc. with frequencies 11,804, 5159, and 4803, respectively. The percentage of frequencies for each of the keywords have also been calculated by keeping the total frequency for the 1000 keywords. Both the frequency and percentage of frequency clearly suggest that most of the work related to proteome has been done in humans and animals. Methodologies such as 2D-gel-electrophoresis, mass spectrometry, matrix-assisted laser desorption-ionization, liquid chromatography, and bioinformatics are the most used ones according to the findings. The density visualization, which presents the density of publications across keywords, also coincides with the fact mentioned above about the keyword frequencies (Figure 3b).

PTC provided annotation information for 29,234 articles out of 33,028 PMIDs subjected for mining. The mining looked at 10 types of bioconcepts (classes), which are CellLine, Chemical, Disease, DNAMutation, Gene, Genus, ProteinMutation, SNP, Species, and Strain. A total of 322,026 bioconcepts were extracted falling under the above classes (Table 3). The bioconcept class Species had the highest number of bioconcept annotations, which is 98,340, followed by Chemical, Disease, Gene, etc., to the class Strain at the last place with 8 annotations. The highest number of unique bioconcept annotations were obtained in the case of the class Gene, however, there is no bioconcept without ID. On the other hand, the class Chemical had 3476 unique bioconcept annotations with 12,062 numbers of bioconcept annotations without ID. Annotations without IDs were obtained only in the case of CellLine, Chemical, and Disease.

**Figure 4:**
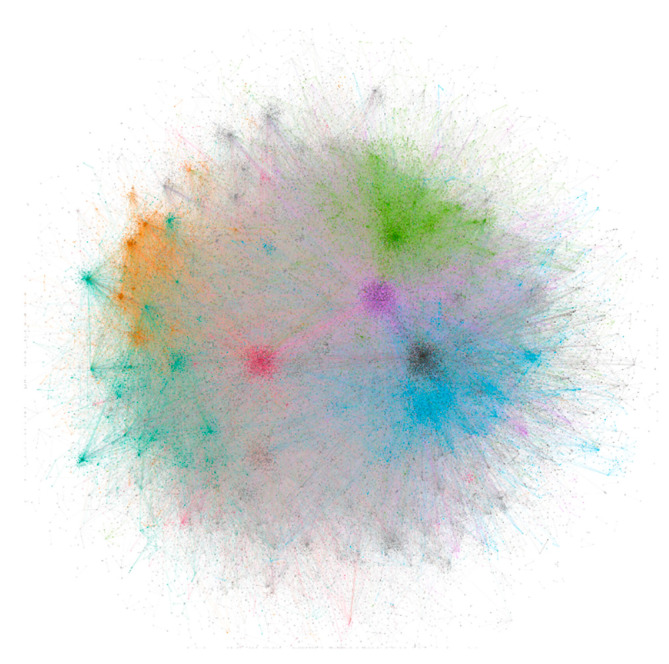
The PMID-bioconcept ID network created on R and visualized on Gephi using OpenOrd layout algorithm. The network was allowed to simulate to attain a stable layout.

The PMID and bioconcept ID association network was created and saved as an image file, which is shown in Figure 4. The network had 52,250 nodes and 148,688 edges with all parallel edges merged by summing the weights. Since the clustering coefficient was not able to provide a clear distinction between clusters, the modularity classes were chosen to color the nodes. The average degree and average weighted degree were 5.691 and 12.326, respectively. The modularity was calculated to be 0.61. Both the highest degree and the highest weighted degree was found for the node Species_9606, which is human with values 11,600 and 28,144, respectively. The PMID:28325852 was found to be the article from which the highest 43 number of bioconcepts were extracted. These 43 bioconcepts consisted of 32, 8, 2 and 1 bioconcepts under the classes Gene, Chemical, Disease and Species, respectively.

**Figure 5:**
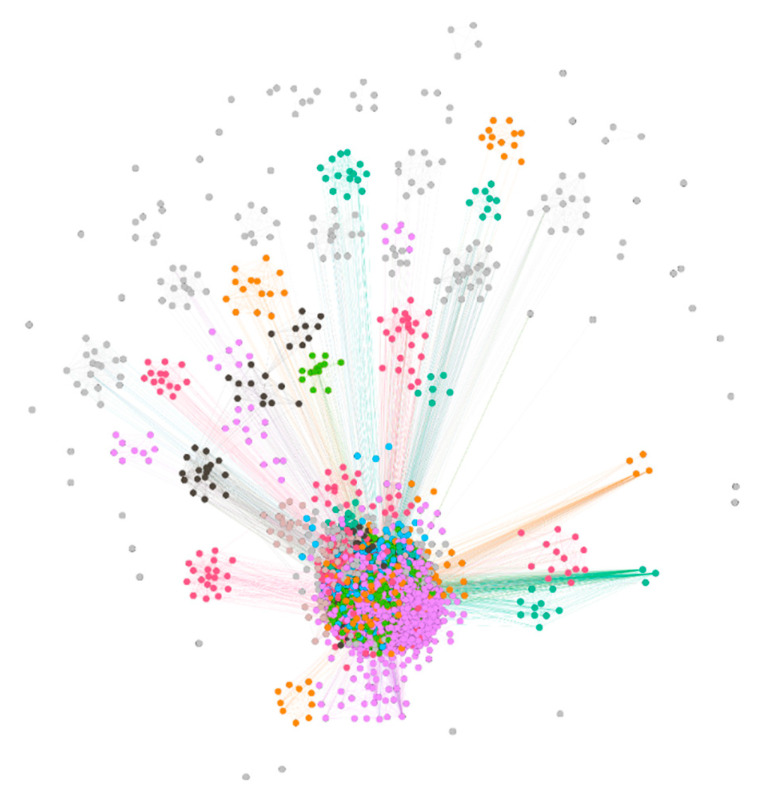
The bioconcept–bioconcept network created on R and visualized on Gephi using OpenOrd layout algorithm. The network was allowed to simulate to attain a stable layout.

The PMID-bioconcept ID network was not very informative about the association of the different bioconcepts, which compelled the creation of networks for the association between all bioconcepts. The co-occurrence of bioconcepts was considered as edges between them, and a network was constructed (Figure 5). The network consisted of a total of 22,864 bioconcept annotations as nodes and 296,799 edges between them. The average degree, average weighted degree and modularity were calculated to be 25.962, 78.3 and 0.355, respectively. The node Species_9606, which represents humans was found to have both the highest degree of 11,837 and the weighted degree of 107,364. The edges suggest that the nodes Species_9606 and Disease_MESH:D009369 had the highest number of associations, which is 4056. The node Disease_MESH:D009369 stands for cancer disease.

**Figure 6:**
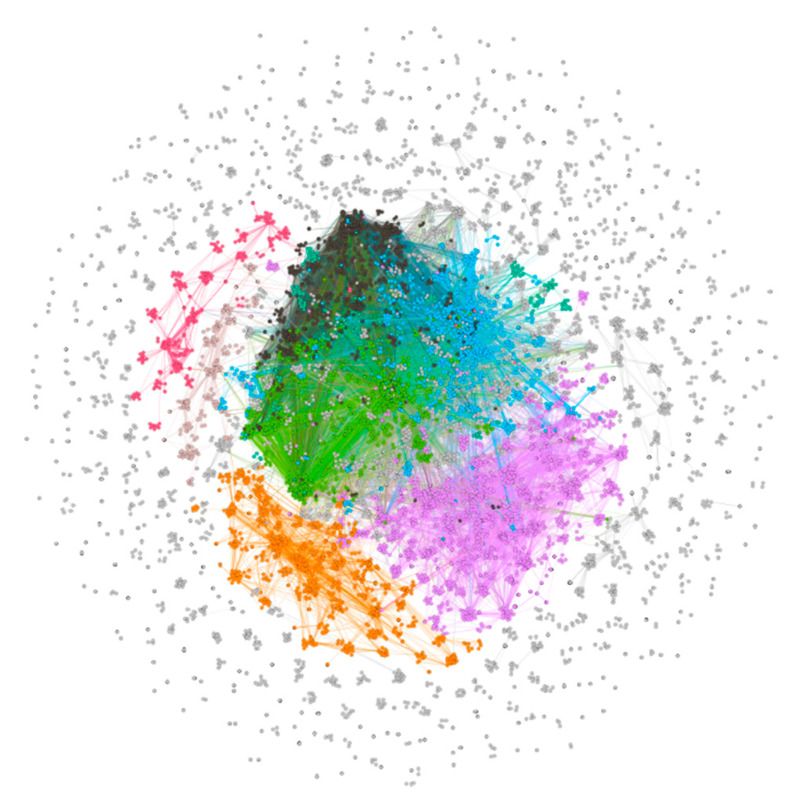
The gene–gene network extracted from the bioconcept–bioconcept network. The network was visualized on Gephi using OpenOrd layout algorithm and allowed to simulate to attain a stable layout. The node color depends on the modularity and the node size ranges from 20 to 50 based on the weighted degree.

Further, the extraction of interactions of bioconcepts belonging to only the Gene class has been presented as a new network (Figure 6). A total of 11,100 unique genes were connected with 64,834 edges with modularity of 0.647. The degree ranged from 1 to 425 with an average degree of 11.682, whereas the weighted degree ranged from 2 to 1414 with an average weighted degree of 25.484. The minimum weighted degree being 2 with the minimum degree 1 suggests that for each gene-gene co-occurrence, the frequency is at least 2. The edges suggest that the nodes Gene_2475 and Gene_207, which stand for the genes mTOR and AKT, respectively, had the highest 64 number of associations. This co-occurrence was obtained from 32 articles that were considered in the current study. The top 10 genes were selected based on the highest weighted degree and associated information from the network were compiled into a table (Table 4). The topmost gene was p53 with a connection to 425 number of genes with the highest weighted degree of 1414. The highest frequency of p53 connections was 52 with the gene MYC. The genes AKT and mTOR made their places in the top 10 with third and tenth positions, respectively.

## 4. Discussion

The present piece of research is a demonstration of the efficiency of text mining to bring out holistic meanings out of unstructured text data. The upshots of the current work revolve around the extraction of scientific information along with findings towards basic bibliometric measures. There has been an upsurge of similar reports where researchers are trying to make meaning out of scientific content and develop new data analytics as well as visualizations [3,4]. Numerous efforts are being made for the development of efficient strategies and methodologies to analyze the most unstructured data which is text. The need for text mining is well understood in many senses. Though there is a number of benefits of using text mining, here are a few significant facts. The first one would be to handle the Big Data issue with respect to the lack of manual handling of the massive data. The second important aspect is to assimilate the information scattered across various domains to come up with a combined meaning, thereby connecting the missing dots. The next and most important aspect is to build robust and powerful tools so that the automated text mining can be performed. Researchers can make use of these resources to find the research gaps and present their own research outputs in a more illustrative manner. To achieve such a degree of efficiency, data analytic tools such as R and python in combination with techniques like machine learning and deep learning are being employed rigorously.

Proteome and proteomics have been trending topics for almost the last three decades along with other omics related buzz words. A vast number of researchers have tried to use these approaches to answer various biological problems. As a result of which, the exponential growth of reports in the form of scientific articles has been observed. There is enormous number of scientific articles available for proteome research yet to be analyzed to produce significant gist. Previously also, a few studies attempted to look into proteome related articles. The first effort to use text mining in the biomedical domain was in the year 1999 to analyze gene expression data [19]. In this study an internet-based hypertext program, MedMiner, was demonstrated. It used the data from the Weizmann Institute’s GeneCards and PubMed database and mined using Perl scripts on a web server resulting in annotated genes related to the user’s query. A study by Blaschke et al. used sets of pre-specified protein names and verbs to define the interactions between proteins nearly accurate to that of manual curation [20]. The text mining techniques improved over time to be more and more accurate as well as efficient for numerous bioconcept types. Tools such as SemRep, BANNER, PubTator, and many more provided effective ways to information extraction from scientific text [21,22,23]. A resource named iProLINK (integrated Protein Literature INformation and Knowledge) developed by the Protein Information Resource (PIR) focused to provide curated and better-structured data which can be used for effective text mining [24]. A systematic review was reported on protein abundances in human skeletal muscle responses aiming at patho-physiological and physiological adaptations associated with obesity, impaired glucose tolerance (IGT), or T2DM and exercise training, respectively [25]. The study was more of a meta-analysis; however, the selection of articles and the review process was a systematic one. There are many other similar studies that have been reported in recent years, however, the aims of these reports are very diverse and different from what the current article is trying to offer.

The present analysis on PubMed reports furnished with NER to extract important entities such as genes, mutations, SNPs, etc., up to 10 classes, which were provided by PubTator, a powerful tool with pre-defined annotation on PubMed records. Though there are many other powerful entity mining tools, the current work uses PubTator as only PubMed data was considered. A total of 23,012 unique bioconcepts were extracted containing certain IDs as well as class tags. The highest number of unique bioconcepts was 12,133 for the Gene class, whereas Strain contained the lowest value 3.

Further extension of the analysis pitched in the construction of co-occurrence graphs for bioconcept–bioconcept associations. To get a flavor of biological insight, the gene–gene co-occurrence network was built. Gene–gene networks are a very popular area of research that has been obtained by various methods. There is co-expression network that is immensely informative concerning the system-wide understanding of regulatory circuits [26,27]. Gene–gene co-expression networks are generally built upon the expression datasets generated from experiments such as microarray, next-generation sequencing, etc. There are even many studies where meta-analyses have been performed to combine the available high-throughput data. Currently, the co-expression network is the most insightful network type, however, it does not look into the assimilation of scattered information or even looking at the studies that are not high-throughput and also these are huge cost associated [28]. Literature mining to obtain a gene–gene co-occurrence network helps in these areas where no other methodologies look. There are also significant works done to construct gene–gene interaction networks by mining texts. STRING database is very well-known for providing the functional interactions between expressed proteins for several organisms [29]. It uses text mining as one of the methods to capture the interaction information from PubMed abstracts. A study in 2016 developed a gene–gene interaction extractor and captured interactions and the relations were further validated using DeepDive, a trained system [30]. The study was carried on 100,000 full-text articles and able to extract 3356 unique gene–gene interactions. A report by Al-Aamri and colleagues presented a text mining approach for gene–gene interaction networks for the human genome, further extending to the identification of disease-specific genes in the networks [31].

Another important aspect is to mine keywords that are frequent, which eventually leads to research area categorization. The study shows that most of the proteome related works have been done in case of the humans. This fact was established during keyword mining as well as bioconcept co-occurrence networks. The frequent keywords also suggested that the most frequent methodologies are 2D-gel-electrophoresis, mass spectrometry, matrix-assisted laser desorption-ionization, liquid chromatography, computational biology which are the most common ones in the case of proteome and proteomics research [32,33,34,35].

The upshots of the study also provided a bit about the publication patterns. The publication trend was not a straight growth in nature. There are certain drops and ups throughout different years making the graph more like a zigzag fashion (Figure 1). A total of 8 continuous years starting from 2012 to 2019 produced more than 2000 articles with 2014 at the top with 2380 articles. There is a significant drop in the year 2020 than of 2019 which is from 2232 to 1458 in number. The interesting fact is that though the year 2021 was not considered while searching the plot, it shows that too with 48 articles. The reason is that the publication years have been considered for plotting. There may also be another reason for the lower number of publications during 2020, which is that sometimes it takes time for journals to make it available on PubMed. There is also another possibility that is not at all technical, but may have a huge impact. It is a pandemic situation due to COVID-19 and the experimental works have been momentously hampered.

On the other hand, the difference between the total number of PMIDs collected and that under subheadings is because some of the articles have not been classified under Mesh terms yet. Most of the subheadings not participating in the intersections with respect to containing common PMIDs may happen because the actual subheadings are largely different from each other. The irregularities in the collection of data, as well as the structuredness of text data, make it difficult to come up with stable output, however, so much information is produced, which is near to impossible to extract with manual efforts. There are certain limitations to this kind of endeavors, which sometimes comes as a gigantic blockage in performing effective text mining. To start with, the unavailability of data or restricted access to data is the largest issue to conduct these kinds of research. The next big issue is the available data analytic approaches. For example, NLP is considered as one of the most efficient approaches, still, it is in its developing stage and lacks to a certain extent while mining scientific texts. The current study only considers the PubMed database and that of the titles and abstracts. This must have resulted in losing lots of information, especially bioconcepts, considering the fact that the full text contains detailed information.

**Figure 7:**
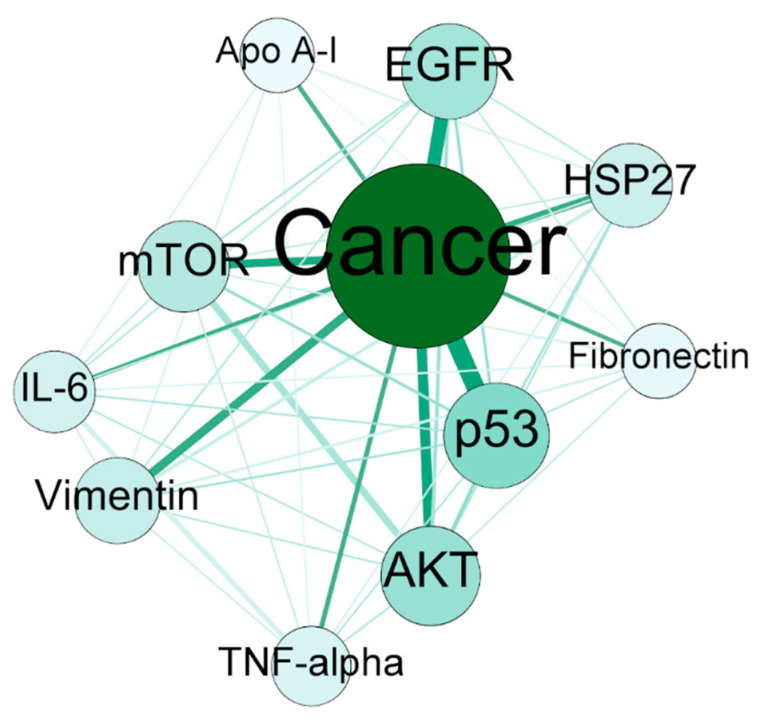
The network for key nodes mined for biological insight. The top 10 genes and the most important disease, which is cancer, were taken as selected nodes, and a network was derived from the bioconcept–bioconcept co-occurrence network.

The current study aimed at providing lots of information along with defining the scope of text mining to serve in many areas. Though there was no certain aim towards specific bioconcept or keywords, the important outcomes of the study make a scientific story. Cancer has been found to be one of the most important nodes based on edge properties along with Human as one of the most frequent keywords. Additionally, almost all of the top genes from gene–gene co-occurrence network (see Table 4) are known to be cancer related genes. Further, for instance of biological insight into the study, the network between Cancer and the top 10 genes clearly mentions the strong associations (Figure 7). Cancer is the most important node with a degree of 10, which suggests that all the top 10 genes are connected to it. The weighted degree of the node is 405, which is extremely high considering the network only contains 11 nodes. The p53 was found to be the most co-occurred gene with Cancer, which is well-known and purely expected. Many studies have established the fact that p53 is the most frequently altered tumor-related gene and cross talks with the stress response mechanisms, thereby transactivating several genes involved in the induction of apoptosis [36,37,38]. Studies have shown that the evolutionarily conserved nature of AKT and mTOR pathways playing vital roles during cell growth and proliferation [39]. Both of these pathways are downregulated by the p53 gene by upregulating some of the negative regulators such as IGF-BP3, PTEN, TSC2, AMPK β1, and Sestrin1/2. The AKT and mTOR are also predominantly associated with Cancer as well as with each other in the network shown. The second most important node in the context of association with Cancer is EGFR. EGFR is also a well-known driver of tumorigenesis in case of several types of cancers such as lung, breast and brain cancer [40]. The EGFR is the target gene for many scientists trying to find a therapy for cancer. The association between EGFR and AKT is quite strong in the network. Vimentin, HSP-27 and Fibronectin are the three important potential targets in the case of cancer therapy [41,42,43]. The IL-6 has been reported to be overexpressed in most types of cancers and regulates almost all of the important signaling pathways [44]. The TNF-alpha has also been reported to be one of the master regulators of tumor-associated inflammation along with IL-6 [45]. The multifunctional protein Apo A-I, a major protein component of high-density lipoprotein (HDL), has been reported as therapeutic against cancers [46]. The above observation suggests that the proteome analysis in the field of cancer is quite abundant.

## 5. Conclusions

The present study tried to provide a well-organized way of mining bioconcepts, commonly also known as entities, from scientific articles. There are many efficient ways of doing the same, especially if a specific area or domain of interest is determined at the beginning of a study. However, this method of performing text mining would be of interest to many researchers since there is not always a pre-determined aim associated with all the studies. In addition, this would definitely help to perform a systematic review on the research areas or fields of interest. The research article as well as other scientific literature are exponentially growing on various public domains and so are the data analytic techniques getting more and more powerful day by day. The research community, especially data scientists, are aiming at mining the scientific texts up to an extent where it would be equivalent or near equivalent to the manual annotation, but with a pace that humans could not do. The upshot of the present study provided several publication patterns as well as many important features to infer into scientific endeavors in the field of proteome research. This type of analysis is never ending, for example, one could be interested in looking at species–species or disease–disease or gene–disease, or similar co-occurrence networks. The same procedure could be followed to develop any of the above-mentioned networks. There are also a good number of projects working on providing real meaningful relationships between two bioconcepts, and the future work will be to create such type of networks where the co-occurrence could be replaced with more authentic regulatory relationships as well as providing specific types of networks such as gene–disease networks.

## Figures and Tables

**Table 1:** Overview of basic findings with respect to scientometrics analysis.

Parameter	Value
Year range	1992–2021
Highest publication year	2014
Lowest publication year	1992
Total number of journals	1745
Most frequent journal	Proteomics
Highest no of publications in a journal	2500
Top publishing country	United States

**Table 2:** Top 20 frequent Mesh keywords mined from selected articles using VOSviewer. The frequency and percentage of frequency were calculated by considering only the top 1000 keywords in the list.

Sl. No.	Term	Frequency	Frequency (Percentage)
1	proteome	32,273	10.228
2	humans	15,686	4.971
3	proteomics	13,016	4.125
4	animals	11,804	3.741
5	electrophoresis, gel, two-dimensional	5159	1.635
6	mass spectrometry	4803	1.522
7	male	4654	1.475
8	female	4474	1.418
9	tandem mass spectrometry	3615	1.146
10	mice	3419	1.084
11	gene expression profiling	3105	0.984
12	spectrometry, mass, matrix-assisted laser desorption-ionization	2984	0.946
13	chromatography, liquid	2978	0.944
14	amino acid sequence	2969	0.941
15	signal transduction	2947	0.934
16	bacterial proteins	2816	0.892
17	molecular sequence data	2770	0.878
18	computational biology	2372	0.752
19	biomarkers	2262	0.717
20	proteins	2219	0.703

**Table 3:** Frequency of bioconcepts under all 10 classes mined from the articles using PubTator.

Bioconcept Class	Total No. of Bioconcepts	No. of Unique Bioconcept Annotations	No. of Bioconcepts with No ID
CellLine	318	74	35
Chemical	80,803	3476	12,062
Disease	76,456	2672	3530
DNAMutation	118	55	0
Gene	65,533	12,133	0
Genus	33	16	0
ProteinMutation	377	197	0
SNP	40	36	0
Species	98,340	4350	0
Strain	8	3	0

**Table 4:** Top 10 genes based on the weighted degree in the gene–gene co-occurrence network.

Sl. No.	ID	Name	Weighted Degree	Degree	Most Interacting Node (ID|Name)	Frequency of Most Interacting Node
1	7157	p53	1414	425	4609|MYC	52
2	7431	Vimentin	1174	398	3315|HSP27	22
3	207	AKT	1118	325	2475|mTOR	64
4	3569	IL-6	972	289	3576|IL-8	42
5	7124	TNF-alpha	932	314	3569|IL-6	42
6	1956	EGFR	916	303	2064|HER2	28
7	335	Apo A-I	878	244	3240|Haptoglobin	32
8	2335	Fibronectin	862	333	7431|Vimentin	14
9	3315	HSP27	860	281	7431|Vimentin	22
10	2475	mTOR	820	277	207|AKT	64
